# Development and Validation of Proactive Health Capacity Measurement Scale for Older Adults in China

**DOI:** 10.1093/geroni/igaf122.2383

**Published:** 2025-12-31

**Authors:** Mengya Zhu, Qianhui Pan, Yexuan Xiao, Nan Jiang

**Affiliations:** Tsinghua University, Beijing, Beijing, China (People’s Republic); Tsinghua University, Beijing, Beijing, China (People’s Republic); Tsinghua University, Beijing, Beijing, China (People’s Republic); Tsinghua University, Beijing, Beijing, China (People’s Republic)

## Abstract

Objective Proactive health capacity refers to individuals’ ability to actively engage in and manage their own health. However, existing proactive health capacity measurement tools have not been specifically developed for older adults. This study aims to fill the gap in China, which can guide the development of targeted self- health management interventions. Methods The scale was developed following COSMIN guidelines. Initially, an item pool was created through literature review, and the scale was developed using Delphi expert consultation. The items were categorized based on Self-Management Theory. A cross-sectional survey of 390 older adults (aged≥60) in China was conducted to refine the scale, employing exploratory factor analysis (EFA), followed by reliability and validity testing. Results After two rounds of Delphi consultation, a scale with 5 dimensions and 26 items was developed. Familiarity coefficients were 0.80 and 0.77, authority coefficients were 0.86 and 0.84, and Kendall’s W values were significant (P < 0.05). EFA (n = 390) showed a KMO of 0.844, with two items removed, yielding a final scale of 5 dimensions and 24 items (Cronbach’s α = 0.911). The dimensions included health problem-solving, self-determination, access and utilize resources, communication and action planning capacity. Discussion The PHCMS enables clinicians to assess older adults’ key self-management capacity, thus guide personalized interventions, evaluate intervention effectiveness, and inform public health strategies. Ultimately, this tool may improve older adults’ well-being by promoting proactive engagement in health and preventive care.

